# An apicoplast-resident folate transporter is essential for sporogony of malaria parasites

**DOI:** 10.1111/cmi.13266

**Published:** 2020-10-08

**Authors:** Francois Korbmacher, Benjamin Drepper, Theo Sanderson, Peer Martin, Thomas Stach, Alexander G. Maier, Kai Matuschewski, Joachim M. Matz

**Affiliations:** 1 Department of Molecular Parasitology, Institute of Biology, Humboldt University, Berlin, Germany; 2 Research School of Biology, The Australian National University, Canberra, Australia; 3 Malaria Biochemistry Laboratory, The Francis Crick Institute, London, UK

**Keywords:** apicoplast, folate, malaria, membrane transport protein, *Plasmodium*, sporogony

## Abstract

Malaria parasites are fast replicating unicellular organisms and require substantial amounts of folate for DNA synthesis. Despite the central role of this critical co-factor for parasite survival, only little is known about intraparasitic folate trafficking in *Plasmodium*. Here, we report on the expression, subcellular localisation and function of the parasite’s folate transporter 2 (FT2) during life cycle progression in the murine malaria parasite *Plasmodium berghei*. Using live fluorescence microscopy of genetically engineered parasites, we demonstrate that FT2 localises to the apicoplast. In invasive *P. berghei* stages, a fraction of FT2 is also observed at the apical end. Upon genetic disruption of *FT2*, blood and liver infection, gametocyte production and mosquito colonisation remain unaltered. But in the *Anopheles* vector, *FT2*-deficient parasites develop inflated oocysts with unusual pulp formation consisting of numerous single-membrane vesicles, which ultimately fuse to form large cavities. Ultrastructural analysis suggests that this defect reflects aberrant sporoblast formation caused by abnormal vesicular traffic. Complete sporogony in *FT2*-deficient oocysts is very rare, and mutant sporozoites fail to establish hepatocyte infection, resulting in a complete block of parasite transmission. Our findings reveal a previously unrecognised organellar folate transporter that exerts critical roles for pathogen maturation in the arthropod vector.

## 1 Introduction

Folate is a vital co-factor that is required for one-carbon transfer reactions, mostly during DNA synthesis. The importance of folate metabolism for *Plasmodium* parasites is evident from the past therapeutic exploitation of the biosynthetic enzymes of this pathway for the treatment of chloroquine-resistant malaria (Doberstyn, Hall, Vetvutanapibul, & Sonkon, 1976; Harinasuta, Viravan, & Reid, 1967). The active ingredients of the antifolate combination drug Fansidar, pyrimethamine and sulfadoxin, synergistically inhibit the parasite’s dihydrofolate reductase-thymidylate synthase (DHFR-TS) and dihydropteroate synthase enzymes, respectively, causing rapid parasite clearance upon treatment (Nzila, 2006). Despite the swift and global spread of antifolate resistance, Fansidar is still used for intermittent preventive treatment, especially during pregnancy, which improves maternal and birth outcomes in malaria endemic areas (Esu, Oringanje, & Meremikwu, 2019). The parasiticidal activity of antifolates also extends to gametocyte and liver stages, underscoring the universal essentiality of this co-factor for replicative and transmission stages in the mammalian host (Bray, Burgess, Fox, & Miller, 1959; Friesen, Borrmann, & Matuschewski, 2011; Kone et al., 2010; Most, Herman, & Schoenfeld, 1967; Shute & Maryon, 1954).

Much less is known about the parasite’s folate requirements during oocyst development and sporulation. *Plasmodium* oocysts are embedded in the basal lamina of the *Anopheles* mosquito midgut, where they grow into large encapsulated spheres with a diameter of ~50 μm. Oocysts undergo several rounds of nuclear division followed by a concerted and tightly synchronised cytokinesis programme, which results in the formation of thousands of sporozoites. The process of sporulation begins with the detachment of the plasma membrane from the oocyst capsule. By continued vesicle fusion, the plasma membrane forms large fissures to ultimately yield isolated islands of parasite cytoplasm, the sporoblasts (Burda et al., 2017; Sinden & Strong, 1978; Terzakis, Sprinz, & Ward, 1967; Vanderberg, Rdodin, & Yoeli, 1967). Here, parasite organelles align below the plasma membrane from where the sporozoites emerge through a budding mechanism (Burda et al., 2017; Stanway, Witt, Zobiak, Aepfelbacher, & Heussler, 2009; Vanderberg et al., 1967). Upon protease-mediated rupture of the oocyst capsule (Aly & Matuschewski, 2005), these slender and highly motile parasite stages migrate to the salivary glands from where they are then transmitted during the mosquito’s blood meal. A recent genetic investigation of folate synthesis and salvage during *Plasmodium* life cycle progression suggested that the parasite’s folate requirements might be rather modest during mosquito infection, when compared to the pronounced folate-dependency of mammalian-infective parasite stages (Matz et al., 2019). Nonetheless, the necessity to increase the DNA content by >1,000-fold during oocyst development indicates that folate must also be required during infection of the *Anopheles* vector.

For endogenous folate biogenesis, the parasite synthesises or imports the central precursor *para*-aminobenzoate (*p*ABA) to fuse it with a pterin moiety derived from purine metabolism, followed by poly-L-glutamylation (Müller & Hyde, 2013). Evidence from other cell systems indicates that folate metabolism is highly compartmentalised, including reaction sequences in the cytosol, mitochondrion, plastid, vacuole and nucleus (Hanson & Gregory 3rd., 2011; Tibbetts & Appling, 2010). Malaria parasites harbour two endosymbiotic organelles, the mitochondrion and a relic non-photosynthetic plastid, known as the apicoplast (Ralph et al., 2004; van Dooren, Stimmler, & Mcfadden, 2006). Folate and/or intermediate metabolites are expected to enter these organelles; however, the identity of the involved transport proteins remains unknown.

To date, two folate transporters, called FT1 and FT2, have been identified in the *Plasmodium* genome datasets. Both proteins contain two domains with high similarity to the *Leishmania* biopterin transporter 1 (BT1), a defining signature of BT1 transmembrane proteins (PFAM: PF03092; Lemley et al., 1999; Myler, Lodes, Merlin, de Vos, & Stuart, 1994), which belong to the major facilitator superfamily (MFS). FT1 and FT2 facilitate the transport of folate and folate precursors when expressed in *Xenopus laevis* oocytes and restore normal growth in *Escherichia coli* bacteria deficient in folate biosynthesis and salvage (Salcedo-Sora et al., 2011). Using polyclonal antisera of uncharacterised specificity, the *P. falciparum* orthologues of FT1 and FT2 were shown to localise to the parasite periphery, tentatively implicating them in folate salvage rather than intraparasitic folate shuttling (Salcedo-Sora et al., 2011).

Using a combination of experimental genetics and quantitative live cell microscopy, we demonstrate here that *P. berghei* FT2 is an apicoplast-resident membrane transport protein required for *Plasmodium* oocyst development and the production of infective sporozoites.

## 2 Results

### 2.1 Evolutionary origin and domain architecture of *Plasmodium* folate transporters

**Figure 1:**
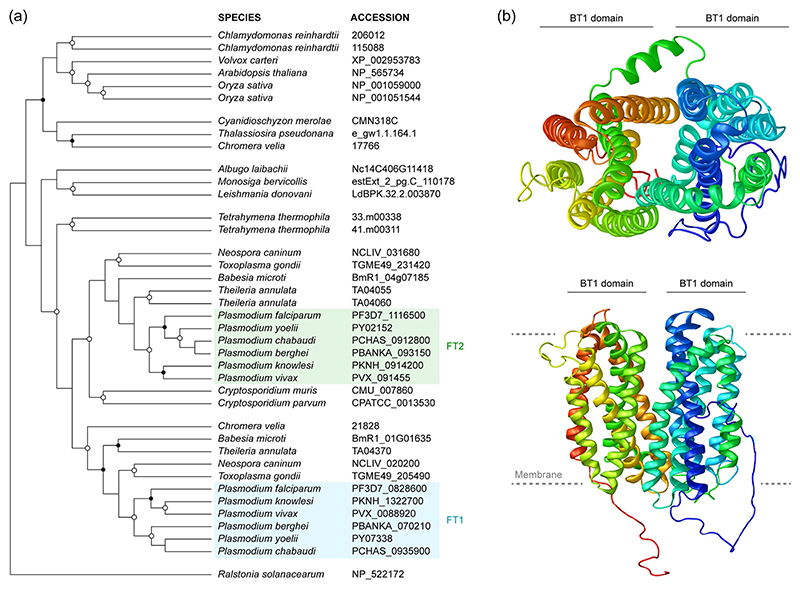
BT1-family transport proteins in *Plasmodium*. (a) Phylogenetic analysis reveals duplication of an ancestral folate transporter in apicomplexan parasites. Shown is a maximum likelihood tree of 38 biopterin transporter 1 (BT1) family proteins from apicomplexan parasites, related Chromalveolata and from additional representative eukaryotic species, as well as one related protein of the major facilitator superfamily (MFS) of bacterial origin. Open circles, nodes with bootstrap values of more than 90%. Closed circles, nodes with values of 65–90%. The *Plasmodium* folate transporters FT1 (blue) and FT2 (green) are highlighted. (b) Structure homology modelling of *Plasmodium berghei* FT2 indicates the signature MFS architecture, characterised by two separate domains (BT1 domains) surrounding a translocation centre, each containing six transmembrane helices. Shown are top (top) and side views (bottom). The phospholipid bilayer is indicated by grey dashed lines. The model was obtained with I-TASSER (Yang & Zhang, 2015) using the *Escherichia coli* YajR transporter structure (PDB ID: 3wdoA; Jiang et al., 2013) as the homology template. Coverage, 0.875; *Z*-score, 3.70; *C*-score, −1.12; TM-score, 0.832

We initiated our analysis by investigating the evolutionary origin and history of the two *Plasmodium* folate transporters, using maximum likelihood phylogenetic reconstruction. As expected, BT1 proteins occur over a broad phylogenetic spectrum of organisms with a history of endosymbiosis, supporting the previously suggested cyanobacterial origin of the BT1 protein family (Klaus et al., 2005; Figure 1a). The two *Plasmodium* folate transporters are both conserved across a range of apicomplexan species. Together with the appearance of a *Chromera* gene in the FT1 clade, this suggests that both *Plasmodium* folate transporters are the result of an ancestral gene duplication preceding the apicomplexan radiation.

Since there are currently no structural data available for any BT1 family member, we employed homology modelling to approximate the three-dimensional structure of *P. berghei* FT2, using the experimentally validated structure of an *E. coli* MFS transporter as template. The recovered model indicates that *Plasmodium* FT2 might share the typical structural features of MFS proteins (Figure 1b). According to this prediction, *Pb*FT2 contains two separate domains with six transmembrane helices each, surrounding a translocation centre. Hereinafter, we describe the experimental genetic characterisation of FT2 during life cycle progression of *P. berghei*.

### 2.2 FT2 resides in the apicoplast and exhibits stage-dependent dual localisation

**Figure 2:**
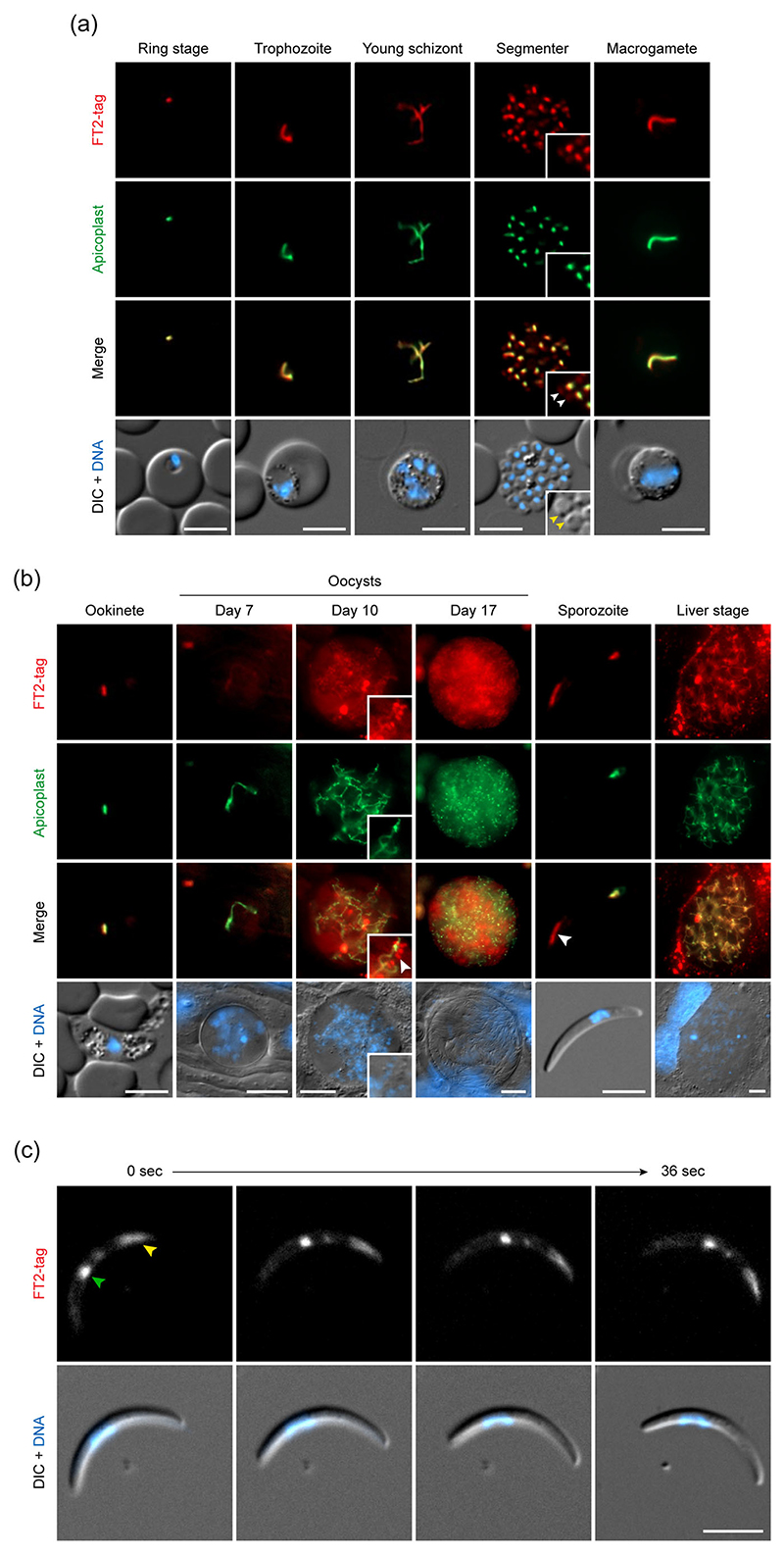
FT2 is an apicoplast protein and broadly expressed during *Plasmodium* life cycle progression. (a, b) Expression of FT2 throughout the *Plasmodium berghei* life cycle. *ft2-tag*_apiGFP_ parasites were imaged live at different stages. Shown are representative images of blood stages (a) and mosquito and liver stages (b), including the fluorescent signals of tagged FT2 (red, first row), the apicoplast marker api-GFP (green, second row) a merge of both signals (third row) as well as a merge of differential interference contrast images (DIC) with Hoechst 33342 nuclear stain (DNA, blue, fourth row). Note that a fraction of FT2 is observed in non-apicoplast structures in merozoites, sporulating oocysts and sporozoites (white arrowheads). Yellow arrowheads, apical prominence in merozoites. Extra-parasitic red fluorescence during the liver stage is due to autofluorescence of host cell vesicular compartments and was also observed in non-fluorescent WT parasites and uninfected host cells. (c) FT2 shows dual localisation to the apicoplast and to the apical end in sporozoites. Shown is a sporozoite recorded during motility, including the signal of tagged FT2 (top) and a merge of DIC with Hoechst 33342 nuclear stain (DNA, blue, bottom). Green arrowhead, apicoplast-localised FT2. Yellow arrowhead, FT2 at sporozoite apex. The dual localisation pattern is representative of all analysed sporozoites. *n* = 90 sporozoites from two independent feeding experiments. Bars, 5 μm for all except oocysts and liver stages (10 μm)

To investigate the subcellular localisation and temporal expression of FT2 in the rodent malaria model parasite *P. berghei*, we generated transgenic parasites expressing endogenous *FT2* fused to an mCherry-3xMyc tag (Figure S1a). Successful modification of the *FT2* locus and expression of the tagged protein were confirmed by diagnostic PCR of genomic DNA and by immunodetection of mCherry in the parasite membrane fraction (Figure S1b,c). Microscopic examination of fluorescently labelled FT2 uncovered a striking organellar localisation throughout *Plasmodium* blood stage development (Figure S1d). The complete lack of organelle branching in female gametocytes was indicative of the apicoplast rather than the mitochondrion (Figure S1d). We therefore generated a second independent parasite line that, in addition to mCherry-3xMyc-tagged FT2, expresses a GFP-fluorescent apicoplast reporter (api-GFP). Live co-localisation analysis revealed a striking overlap of tagged FT2 and api-GFP (Figure 2a,b). mCherry fluorescence was detected in all stages of the *P. berghei* life cycle, together suggesting that FT2 is a broadly expressed membrane transport protein of the apicoplast.

We also detected a small fraction of FT2 at the apical prominence of merozoites, as observed by fluorescence and differential interference contrast imaging (Figure 2a). Similarly, in sporulating oocysts, FT2 was detected in the branching and dividing apicoplast and in additional fluorescent foci (Figure 2b). Inspection of sporozoites revealed that extra-plastidial FT2 localises to an elongated structure at one end of the parasite (Figure 2b). Using time-resolved live fluorescence imaging of gliding sporozoites, this fraction was consistently detected at the apical parasite pole (Figure 2c). Together, our analysis uncovered a striking organellar localisation of *P. berghei* FT2 to the apicoplast and to the apex of invasive parasite stages, with abundant expression during parasite life cycle progression.

### 2.3 Normal blood propagation and sexual differentiation in the absence of *FT2*

**Figure 3:**
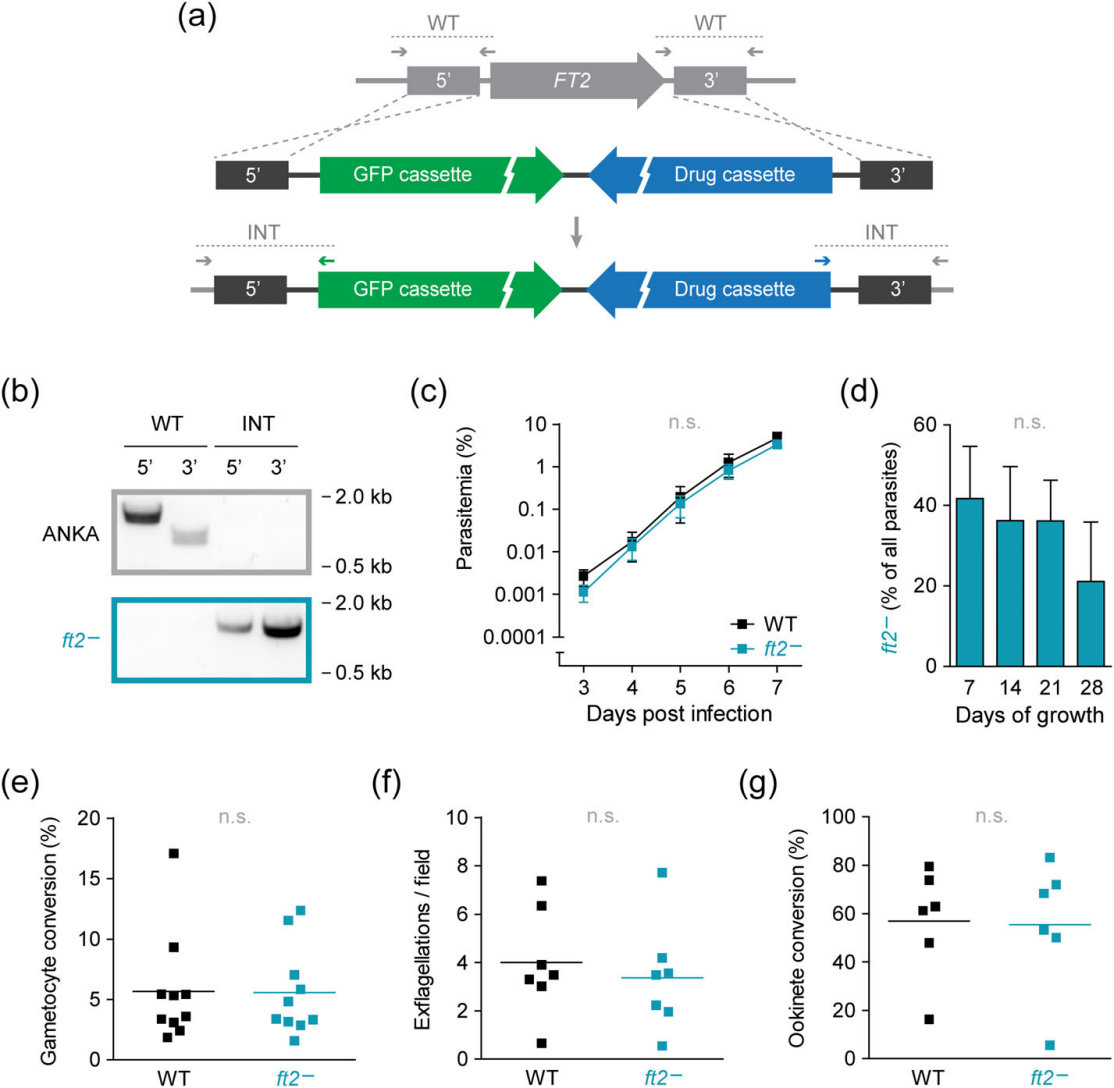
Efficient blood propagation and sexual differentiation in the absence of *FT2*. (a) Replacement strategy for the deletion of *Plasmodium berghei FT2*. The locus was targeted with a replacement plasmid containing the 5′ and 3′ flanking regions, an expression cassette for high-level cytoplasmic GFP fluorescence (green), and the drug-selectable hDHFR-yFcu cassette (blue). Shown are the WT locus (top), the transfection vector (middle) and the recombined locus (bottom). WT-specific and integration-specific (INT) primer combinations are indicated by arrows and expected fragments by dotted lines. (b) Diagnostic PCRs of the WT locus (top) and of the drug-selected and isolated *ft2*^−^ parasites (bottom), using the primer combinations depicted in (a). Note the generation of a second independent *FT2* loss-of-function mutant expressing fluorescent markers of the apicoplast and mitochondrion (see Figure S2a,b). (c, d) Efficient parasite proliferation in the murine bloodstream. (c) Intravital competition assay. Equal numbers of mCherry-fluorescent Berred WT and GFP-fluorescent *ft2*^−^ blood stage parasites were co-injected intravenously into mice and peripheral blood was analysed daily by flow cytometry (Matz, Matuschewski, & Kooij, 2013). Mean values (±*SD*) are shown. n.s., non-significant; two-way ANOVA; *n* = 3. (d) Serial passage of infected blood reveals no significant fitness loss of asexual *ft2*^−^ blood stage parasites. Co-infected blood, as described in (c), was serially transferred into naïve mice after 1 week of infection and the ratio of WT and *ft2*^−^ blood stages was determined by flow cytometry. Mean values (±*SD*) are shown. n.s., non-significant; one-way ANOVA; *n* = 3. (e–g) Normal sexual differentiation in the absence of *FT2*. 10^7^ WT or *ft2*^−^ blood stage parasites were injected intravenously into mice and peripheral blood was analysed 3 days later for gametocyte conversion (e), exflagellation (f) and ookinete conversion (g). Shown are the percentages of mature gametocytes among all blood stages, as identified in Giemsa-stained thin blood films, exflagellation centres per microscopic field and the percentage of fully matured ookinetes among in vitro cultivated P28-positive parasites. Shown are individual data points as well as mean values (bars). n.s., non-significant; Student’s *t* test; *n* ≥ 6

The distinct spatiotemporal expression of *FT2* prompted us to investigate the consequences of *FT2* deletion. Using a replacement strategy based on double homologous recombination, we generated and isolated *P. berghei* parasites lacking the *FT2* gene (Figure 3a). In addition, *ft2*^−^ parasites expressed cytoplasmic GFP facilitating parasite detection by fluorescence microscopy and flow cytometry. Gene deletion was confirmed by diagnostic PCR of the drug-selected and isolated parasite line, highlighting the dispensability of *FT2* for asexual proliferation in the mammalian bloodstream (Figure 3b).

We examined the propagation of asexual *ft2*^−^ parasites during blood infection with an intravital competition assay (Matz et al., 2013). By co-injecting mCherry-fluorescent Berred WT parasites with the GFP-fluorescent *ft2*^−^ parasites, we followed parasite development in the presence and absence of *FT2* within the same mouse using flow cytometry. We detected no differences in the rate of blood propagation in individual animals, and repeated passage of infected blood to naïve mice did not result in out-competition of the *ft2*^−^ strain by WT parasites (Figure 3c,d). Similarly, the ability to develop fertile gametocytes was not affected by the absence of *FT2*, as indicated by the unchanged sexual conversion rates, exflagellation activity and ookinete formation in vitro (Figure 3e–g). Together, these findings indicate that, despite abundant expression, *FT2* is dispensable for both sexual and asexual *Plasmodium* blood stage development.

### 2.4 *FT2* deficiency causes oocyst swelling and intracellular pulp formation

**Figure 4:**
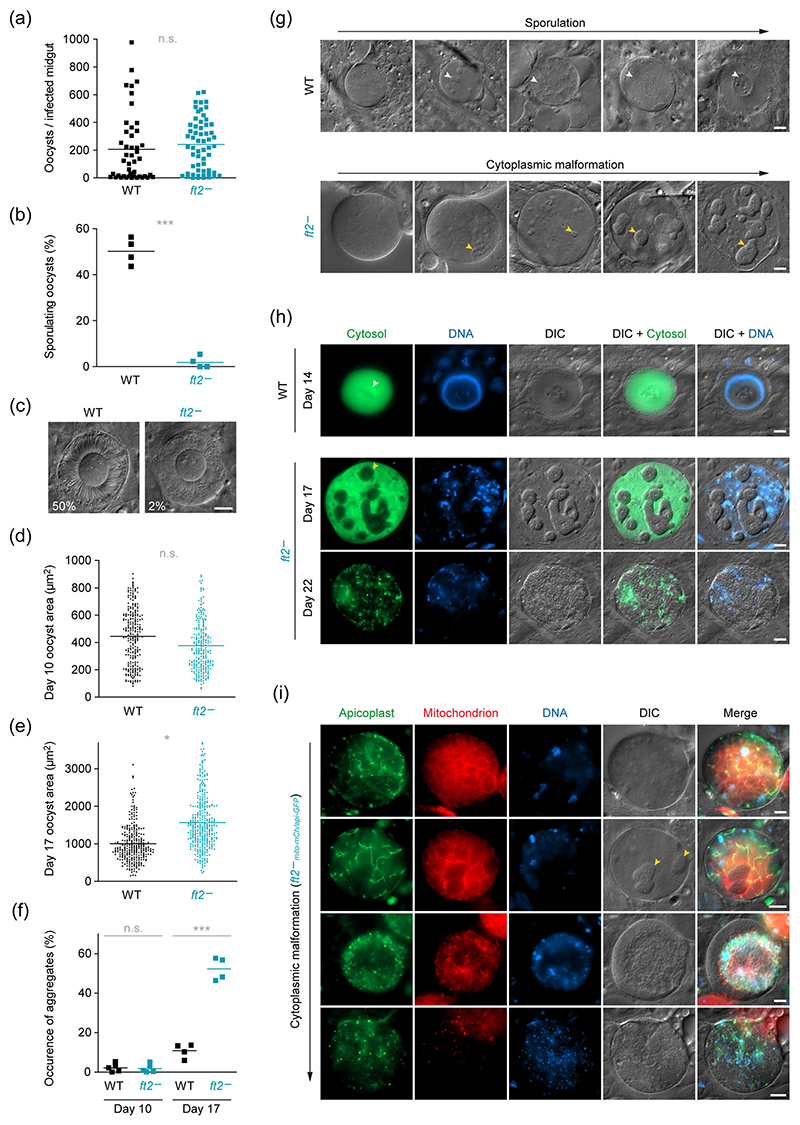
Prominent swelling and cytoplasmic malformations in *FT2*-deficient oocysts. (a) Normal mosquito midgut colonisation in the absence of *FT2*. Shown are oocyst numbers of individual infected midguts and the mean infection intensity (bars) on Day 10 after the blood meal. n.s., non-significant; Student’s *t* test; *n* ≥ 46 midguts from three independent blood meals. (b, c) Oocyst sporulation is severely impaired upon loss of *FT2*. Sporulating oocysts were quantified (b) and imaged by differential interference contrast (DIC) (c) on Day 17 after the blood meal. ***, *p* < .01; Student’s *t* test; *n* = 4 independent blood meals. Percentages indicate the mean sporulation frequency and are derived from analysis of >600 oocysts from >30 infected midguts each. (d, e) Marked swelling of *FT2*-deficient mature oocysts. Infected midguts were imaged live 10 (d) and 17 days after the blood meal (e). Shown is the area occupied by individual oocysts in live fluorescence micrograph. n.s., non-significant; *, *p* < .05; Student’s *t* test; *n* ≥ 254 oocysts from four independent blood meals. (f, g) *FT2*-deficient oocysts form large intracellular malformations. (f) Shown is the percentage of oocysts containing intraparasitic accumulations 10 and 17 days after the blood meal. n.s., non-significant; ***, *p* < .01; Student’s *t* test; *n* = 4 independent blood meals. Percentages are derived from analysis of >600 oocysts from >30 infected midguts each. (g) Depicted are differential interference contrast images of 17 days old oocysts, showing various stages of either sporulation (WT) or cytoplasmic clumping (*ft2*^−^). White arrowheads, sporoblast; yellow arrowheads, intraparasitic accumulations. (h, i) Intracellular malformations displace cytoplasmic GFP and several parasite organelles in *FT2*-deficient oocysts. (h) Shown are images of a sporulating WT oocysts at Day 14 (top) and *ft2*^−^ oocysts on Days 17 and 22 after the blood meal (bottom), including the fluorescent signal of cytoplasmic GFP (green, first column), Hoechst 33342 nuclear stain (DNA, blue, second column), DIC images (third column), and two distinct merges of DIC with either GFP or DNA (last two columns). Note the presence of cytoplasmic GFP within the sporoblast (white arrowhead) and the absence thereof from the intraparasitic accumulations (yellow arrowhead). Also note the complete vesiculation of the *ft2*^−^ oocysts at Day 22. (i) Shown are 17 days old *ft2*^−^_mito-mCh/api-GFP_ oocysts at different stages of cytoplasmic clumping, including the individual signals of api-GFP (green, first column), mito-mCherry (red, second column), Hoechst 33342 nuclear dye (DNA, blue, third column), DIC images (fourth column) and a merge of all channels (fifth column). Note the displacement of apicoplast, mitochondrion and nuclei by the intraparasitic accumulations (yellow arrowheads). Bars, 10 μm

Upon transmission to *Anopheles stephensi* mosquitoes, *ft2*^−^ parasites formed normal numbers of midgut-associated oocysts (Figure 4a). However, by day 17 after the blood meal, only 2% of them had entered sporulation, as compared to 50% of WT oocysts (Figure 4b,c). This striking defect correlated with prominent swelling of the *FT2*-deficient parasites. While the area of *ft2*^−^ oocysts in live fluorescence micrographs was normal at Day 10 after the blood meal, it became 1.6-fold larger than WT by day 17 (Figure 4d,e). Assuming spherical oocyst proportions, this translates into a ~2-fold increase in parasite volume. The inflated appearance of *FT2*-deficient oocysts was further accompanied by the accumulation of extensive intraparasitic structures, which appeared to result from the clumping of cellular components and which were only rarely observed in WT oocysts (Figure 4f). Inspection by differential interference contrast microscopy revealed that these pulps had a rough surface, as if made up by several smaller structures that formed large islands within the oocyst cytoplasm (Figure 4g). We note that these accumulations do not represent the sporoblasts. In WT parasites, individual circular patches are observed upon entering sporogony. However, in normal development, patches appear smoother and more regular than the accumulations in *ft2*^−^ oocysts and represent sites where the plasma membrane has begun to detach from the oocyst capsule to initiate sporoblast formation (Figure 4g). Live fluorescence microscopy of cytoplasmic GFP revealed that the sporoblast of WT (Bergreen) parasites contains parasite cytoplasm (Figure 4h). In marked contrast, the accumulations observed in the absence of *FT2* displaced cytoplasmic GFP (Figure 4h). Similarly, parasite nuclei visualised by Hoechst 33342 nuclear dye did not localise to the accumulations. By Day 22 after the blood meal, *ft2*^−^ oocysts eventually disintegrated, and cytoplasmic and nuclear fluorescence were restricted to punctate speckles within a mass of vesicularised cytoplasm (Figure 4h).

To test whether the cytoplasmic islands might be the result of apicoplast fragmentation, and perhaps accumulating organellar debris, we generated independent dually labelled *FT2* knockout and WT lines, which express the fluorescent organellar markers api-GFP and mito-mCherry (Figure S2a,b). Visualisation of the fluorescent apicoplast and mitochondrion suggested that both endosymbiotic organelles remained intact throughout *Plasmodium* life cycle progression, independent of *FT2* (Figure 4i and Figure S2c). In *FT2*-deficient oocysts, the apicoplast and mitochondrion were highly branched and spread throughout the oocyst cytoplasm and were excluded from the intra-parasitic accumulations (Figure 4i). Interestingly, upon progression of pulp formation, the organelles were pushed towards the centre of the oocyst, suggesting that they were displaced by the growing masses, which appeared to communicate with the oocyst periphery. As with the cytoplasmic and nuclear fluorescence, organellar fluorescence was detected only in small speckles towards oocyst disintegration (Figure 4i).

### 2.5 Pulp formation occurs by defective vesicular traffic in the absence of *FT2*

**Figure 5:**
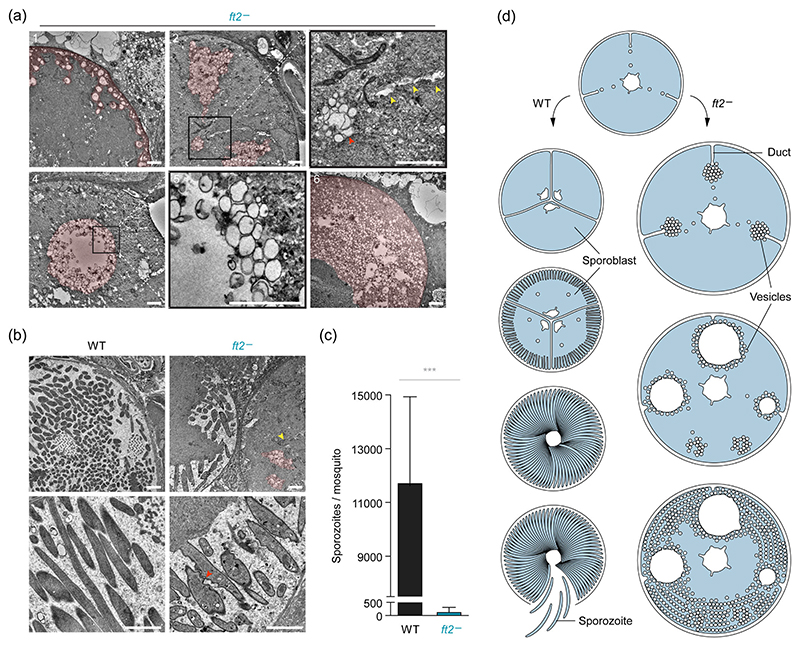
*FT2*-deficient oocysts accumulate single-membrane vesicles. Shown are transmission electron micrographs of 17 days old oocysts. (a) *ft2*^−^ oocysts showing four stages of vesiculation. Initially, vesicles emerge from the oocyst periphery (1) and then form large congregations, which frequently associate with ducts of similar electron density, that appear to emerge from the oocyst periphery (2, 3). Some vesicles appear to fuse to form large cavities covered in single-membrane vesicles (4, 5). Upon progressing vesiculation, vesicles are predominantly found in the oocyst periphery, leaving only a central portion of cytoplasm unaffected (6). Red arrowhead, vesicle accumulation; yellow arrowheads, duct. (b) Occasional budding of malformed *ft2*^−^ sporozoites. Shown are sporulating WT (left) and *ft2*^−^ oocysts (right). Note that vesiculation also occurs in sporulating *ft2*^−^ oocysts. Budding *ft2*^−^ sporozoites showed signs of membrane deformation not commonly observed in WT. Yellow arrowhead, budding sporozoite apex; red arrowhead, membrane deformation. The light pink pseudo coloration highlights vesicle formations. Bars, 2 μm. (c) Quantification of salivary gland-associated sporozoites 21 days after the blood meal. Depicted are averaged sporozoite burdens from ~50 mosquitoes per feeding as well as mean values (bars). ***, *p* < .001; Student’s *t* test; *n* = 4 independent blood meals. (d) Proposed stages of oocyst development in WT parasites (left) or in the absence of *FT2* (right). Upon commencing sporogony, vesicles are trafficked to the oocyst periphery to supply membrane for sporoblast formation, thereby promoting sporozoite production in WT parasites. In *ft2*^−^ oocysts, vesicles fail to undergo coordinated fusion, thus leading to the formation of vesicular patches, which associate with the periphery via membranous ducts and ultimately fuse. Continued trafficking of vesicles to the *ft2*^−^ oocyst periphery leads to the concentration of cytoplasm and parasite organelles in the oocyst centre

Intraparasitic pulp formation in combination with oocyst swelling has not been described yet in *Plasmodium* parasite lines. We therefore investigated the ultrastructural basis of intraparasitic pulp formation and analysed advanced *ft2*^−^ oocysts by transmission electron microscopy (TEM). Close inspection of individual oocysts revealed various stages of vesiculation (Figure 5a). In some parasites, single-membrane vesicles of variable size appeared in close proximity to the oocyst periphery, indicative of initiation of sporoblast formation. In other *ft2*^−^ oocysts, the vesicles had accumulated to form large islands within the cytoplasm, which appeared to be associated with the oocyst periphery by membranous ducts. Some of the islands were in the process of fusing, leading to the formation of large intra-cellular cavities decorated with single-membrane vesicles. At advanced stages of vesiculation, large masses of vesicles and cavities appeared to encircle a central portion of non-vesicularised oocyst cytoplasm, once again highlighting their association with the oocyst periphery.

The vast majority of WT oocysts had undergone sporulation by Day 17, yielding high numbers of encapsulated sporozoites (Figure 5b). In agreement with our light microscopy data, sporulating *ft2*^−^ oocysts were detected only in very few instances and displayed similar vesicle accumulations, albeit lower in frequency and magnitude (Figure 5b). Strikingly, several of the budding *ft2*^−^ sporozoites displayed corrugated membrane deformations and contained a larger number of translucent vesicles than did WT sporozoites, underscoring the imbalance in membrane trafficking that appears to be characteristic of *FT2*-deficient oocysts (Figure 5b). The prominent defects in *ft2*^−^ oocyst sporulation resulted in extremely low numbers of *ft2*^−^ sporozoites (~1% of WT) associated with mosquito salivary glands (Figure 5c). This finding is supported by a recent genetic screen of >1,300 pooled barcoded *P. berghei* knockouts in life cycle progression, which showed a severe loss of the *ft2*^−^ barcode upon transition from midgut oocysts to infected salivary glands (Stanway et al., 2019).

Together, our ultrastructural observations are suggestive of defective sporoblast formation in the absence of *FT2*. Vesicles trafficked to the oocyst plasma membrane no longer undergo coordinated fusion to form the invaginations required for sporoblast genesis, but instead accumulate in large islands, which maintain a loose connection to the oocyst periphery and ultimately fuse. These cytoplasmic vesicle accumulations might effectively deplete membrane material for efficient sporozoite budding and prevent the efficient production of transmissible parasite stages (Figure 5d).

### 2.6 *Plasmodium* life cycle arrest in the absence of *FT2*

**Figure 6:**
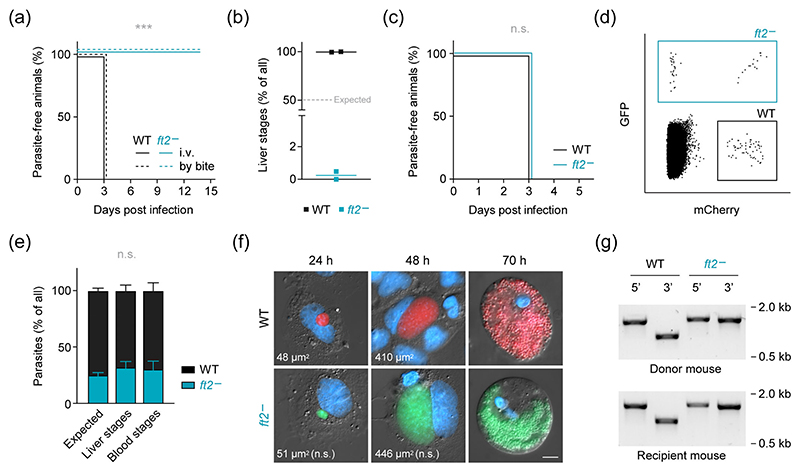
*FT2* is required for formation of infectious sporozoites and dispensable for liver stage maturation. (a) Sporozoites originating from *FT2*-deficient oocysts do not elicit malaria. Kaplan–Meier analysis of time to development of patent blood infection. Naïve mice were intravenously injected with 10,000 WT or *ft2*^−^ sporozoites, or were subjected to bites by mosquitoes that had received blood meals from mice infected with WT or *ft2*^−^ parasites. Peripheral blood was monitored daily by microscopic analysis of Giemsa-stained thin blood films. ***, *p* < .001; Mantel–Cox test; *n* = 3 mice from two independent feeding experiments. (b) Sporozoites originating from *FT2*-deficient oocysts are non-invasive. Huh-7 cells were inoculated with an equal mix of mCherry-fluorescent Berred WT and GFP-fluorescent *ft2*^−^ sporozoites obtained from independent preparations. Shown are percentages of WT and *ft2*^−^ liver stage parasites as quantified by live fluorescence microscopy 48 hr after inoculation. The dashed line represents the 50:50 ratios expected in the case of normal *ft2*^−^ invasion. *n* = 509 parasites from two independent infection experiments. Note that only once a single GFP-fluorescent liver stage was observed. (c–g) Phenotypic rescue during mosquito infection restores *ft2*^−^ sporozoite infectivity and reveals redundant functions of *FT2* during liver stage development. Mosquitoes were fed on mice co-infected with mCherry-fluorescent Berred WT and GFP-fluorescent *ft2*^−^ parasites, leading to cross-fertilisation and phenotypic rescue during mosquito infection. (c) Kaplan–Meier analysis of time to development of patent blood infection. Naïve mice were intravenously injected with 30,000 sporozoites isolated from salivary glands of WT x *ft2*^−^-infected mosquitoes and peripheral blood was monitored daily by flow cytometry. n.s., non-significant; Mantel–Cox test; *n* = 9 mice from three independent feeding experiments. (d) Representative dot plot obtained by flow cytometry on Day 3 after transmission. Double fluorescent parasites are due to inter-chromosomal recombination events during mosquito infection and denote *FT2*-deficient parasites. (e) Quantification of *FT2*-deficient liver stages in vitro and emerging blood stages in vivo after inoculation with the mixed sporozoite population. Expected values reflect the fractions in the case of unaltered parasite fitness and are based on the percentage of observed double-fluorescent sporozoites. Shown are mean values (±*SD*). n.s., non-significant; one-way ANOVA and Tukey’s multiple comparison test; *n* = 3 independent feeding experiments, including data from 1.269 sporozoites, 2.643 liver stages and 751 first-generation blood stage parasites. (f) Representative images of in vitro cultivated WT and *ft2*^−^ liver stages at different times after infection with sporozoites isolated from WT x *ft2*^−^-infected mosquitoes. Shown is a merge of cytoplasmic fluorescence (red, WT; green, *ft2*^−^), Hoechst 33342 nuclear stain (blue) and differential interference contrast images. Values represent the mean area occupied by liver stages in fluorescence micrographs. n.s., non-significant; Student’s *t* test; *n* ≥ 150 liver stages from three independent experiments. Bar, 10 μm. (g) Diagnostic PCR of genomic parasite DNA isolated from the blood of a co-infected donor mouse prior to mosquito feeding (top) and from a recipient mouse after transmission (bottom). Primer combinations are as indicated in Figure 3a

We next tested whether the few produced *ft2*^−^ sporozoites are capable of eliciting patent blood infection and thus subjected naïve mice to the bites of infected mosquitoes or to intravenous injection of isolated sporozoites. While the WT strain consistently caused blood infection 3 days after sporozoite inoculation, mice that had received *FT2*-deficient sporozoites remained blood stage-negative (Figure 6a). We then incubated cultured liver cells with mixed inocula of mCherry-fluorescent WT (Berred) and GFP-fluorescent *ft2*^−^ sporozoites. Among >500 analysed liver stages, we observed a single GFP-fluorescent exoerythrocytic form, indicating that the few sporozoites egressing from *ft2*^−^ oocysts cannot efficiently invade hepatocytes, even when deposited directly onto cultured hepatoma cells (Figure 6b). Together, the impairments in sporozoite production and functionality in the absence of *FT2* cause a complete block of parasite transmission in vivo, highlighting the essential role of this putative folate transporter during host switching.

To further exclude indirect effects due to second site mutations, we generated a complemented parasite line by transfection of an FT2-mCherry-3xMyc fusion protein expression cassette into *ft2*^−^ parasites (Figure S3a–c). Genetic complementation with the tagged version of *FT2* expressed from an independent locus confirmed the signature apicoplast localisation (Figure S3d). Importantly, the combined defects in sporulation and transmission of *ft2*^−^ parasites were completely restored (Figure S3e).

To test whether *FT2* is also required for liver stage maturation, we rescued the sporulation defect by feeding mosquitoes on mice dually infected with green-fluorescent *ft2*^−^ and red-fluorescent WT (Berred) parasites. Cross-fertilisation of both parasite strains in vivo led to the formation of double-fluorescent heterozygous WT x *ft2*^−^ oocysts and thus to the generation of a mixed sporozoite population with individual parasites either containing or lacking the *FT2* gene (Figure S4). Intravenous injection of the haploid sporozoites resulted in the emergence of WT and *FT2*-deficient blood stages 3 days following injection, as indicated by flow cytometry of peripheral blood (Figure 6c,d). Accordingly, infection of cultured liver cells with the mixed sporozoite population resulted in the maturation of both WT and *FT2*-deficient liver stages. Comparison of the fluorescent sporozoite populations with the resultant fractions in liver stages or first-generation blood stages recovered no defects in the absence of *FT2* (Figure 6e). Moreover, size and morphology of *FT2*-deficient parasites remained indistinguishable from WT parasites throughout exoerythrocytic development (Figure 6f). Diagnostic PCR of infected blood confirmed the genotypes of WT and *ft2*^−^ parasites upon transmission of the mixed parasite population (Figure 6g). Together, these findings indicate that the life cycle arrest in the absence of *FT2* is not caused by defective maturation during the liver stage but is rather rooted in the inefficient production of dysfunctional sporozoites within the anopheline vector, underscoring an essential function of the apicoplast transport protein FT2 in *Plasmodium* sporogony.

## 3 Discussion

Here, we have demonstrated that FT2 is a transporter of the apicoplast with essential functions during *Plasmodium* oocyst sporulation. A previous study claimed localisation of the *P. falciparum* FT2 orthologue to the parasite periphery, based on immunofluorescence analysis with polyclonal anti-peptide antisera (Salcedo-Sora et al., 2011). By contrast, we provide genetic evidence for an apicoplast localisation of FT2 in *P. berghei*. Heterologous expression of tagged FT2 restored efficient sporulation and sporozoite infectivity in parasites lacking the endogenous *FT2* gene, demonstrating unaltered functionality of apicoplast-targeted FT2. Although we cannot formally exclude fundamentally different localisation in the related *Plasmodium* species, we consider this possibility unlikely and suggest that the physiological localisation of FT2 in *P. falciparum* requires re-evaluation.

The apicoplast localisation of FT2 disqualifies this protein as a mediator of folate salvage. The other candidate for this molecular pathway, FT1, was shown to be dispensable for asexual blood stage growth in both rodent and human malaria models (Bushell et al., 2017; Zhang et al., 2018). We argue that *Plasmodium* folate salvage might not require a specialised uptake mechanism facilitated by plasma membrane transporters. We have previously shown that the central folate metabolite salvaged by *Plasmodium* blood stages is the precursor *p*ABA (Matz et al., 2019). Owing to its aromatic benzene ring, *p*ABA can diffuse across lipid membranes and thus access the cell passively (Quinlivan et al., 2003). The assumption of a plasma membrane folate transporter essential for *Plasmodium* blood stages might thus be unwarranted. Together with previous biochemical observations (Salcedo-Sora et al., 2011), the distinct localisation of FT2 implies a role in the plastidial transport of folates and/or related pteridine molecules. Further biochemical assays, including metabolic profiling of purified apicoplasts (Botté et al., 2013) from WT and *ft2*^−^ parasites are warranted to solve the molecular gatekeeper function of this intriguing MFS transport protein.

Interestingly, FT2 does not contain a canonical bipartite leader sequence (Foth et al., 2003). This motif, which consists of an ER signal peptide and an apicoplast transit peptide, is characteristic for proteins of the apicoplast stroma and of the innermost plastidial membrane (Mullin et al., 2006; van Dooren, Su, D’Ombrain, & McFadden, 2002; Waller, Reed, Cowman, & McFadden, 2000). By contrast, a transporter of the outermost apicoplast membrane, as well as three additional and yet uncharacterised apicoplast membrane transporters, lack this leader sequence (Lim, Sayers, Goodman, & McFadden, 2016; Mullin et al., 2006; Sayers, Mollard, Buchanan, McFadden, & Goodman, 2018). The absence of a classical apicoplast targeting signal from FT2 might thus signify that FT2 is also inserted into one or several of the outer three membranes of the apicoplast. This would require additional trafficking steps across the remaining membrane(s), perhaps by a solute channel with broad specificity. Nonetheless, how sequential transport across four membranes is achieved remains enigmatic for any apicoplast cargo.

We note that FT2 also localises to the apices of merozoites and sporozoites. Considering that the apicoplast and the invasive organelles intersect with the parasite’s secretory pathway, a secondary localisation of FT2 to the micronemes, rhoptries, dense granules or the apical parasite plasma membrane is conceivable. A mitochondrial localisation appears less likely, partly because the mitochondrion is typically positioned at the posterior sporozoite pole (Frischknecht & Matuschewski, 2017). The exact subcellular localisation of this particular fraction of FT2 and its role in sporozoite functions remain to be determined, however, it is interesting to speculate how this protein distribution might contribute to the defects in sporozoite formation and infectivity. We observed a striking correlation of the dual localisation during sporogony with the timing of life cycle arrest in *FT2*-deficient parasites. Whether the essential functions of *FT2* in sporogony are promoted by the plastidial or extra-plastidial fraction, or both, remains unanswered at the moment. The broad expression pattern of *FT2* suggests that this transporter functions throughout the entire parasite life cycle, yet an essential role is only detected upon productive sporulation in the mosquito vector.

A striking defect in *FT2*-deficient parasites was the vesiculation of oocysts. How an imbalance in pteridine transport leads to this previously unrecognised phenotype remains hypothetical. Since we can exclude defects in apicoplast morphogenesis and integrity, a metabolic imbalance leading to aberrant oocyst growth is more likely. The vital role of the apicoplast during blood infection is biosynthesis of isoprenoid precursors (Yeh & DeRisi, 2011). Recent work has established, that a loss of isoprenoid synthesis causes a halt in protein prenylation, which in turn results in defective vesicular trafficking (Kennedy et al., 2019). In asexual blood stages, this causes fragmentation of the food vacuole and abnormal formation of the inner membrane complex. One could envisage insufficient production of isoprenoid precursors or other metabolites with vital roles in sporogony as a consequence of loss of pteridine transport function, leading to membrane deformations, vesicles, swelling and the general loss of infectivity in *FT2*-deficient sporozoites.

Together, our findings demonstrate the essential role of an apicoplast-resident folate transporter during *Plasmodium* sporogony and provide support for a functional link between organellar folate import and vesicular traffic during the production of the malaria parasite’s transmission stages.

## 4 Experimental Procedures

### 4.1 In silico analyses

For phylogenetic reconstruction, protein sequences were retrieved from EupathDB and OrthoMCL DB using group OG5_130898. We identified additional sequences sharing similarity with *Pb*FT2, using Ensembl Compara. Protein sequences were aligned with MAFFT 7.45 (Katoh & Standley, 2013) and then PhyML 3.0 (Guindon et al., 2010) was used to generate a maximum likelihood tree which was validated by the construction of 104 bootstrap trees.

Structure homology modelling was performed with I-TASSER (Yang & Zhang, 2015). The amino acid sequence of *Pb*FT2 was aligned with the experimentally validated structure of the *E. coli* YajR transporter (PDB ID: 3wdoA; Jiang et al., 2013), which among the available structural homologues shares the closest similarity with FT2.

### 4.2 Experimental animals

This study was carried out in strict accordance with the German ‘Tierschutzgesetz in der Fassung vom 22. Juli 2009’ and the Directive 2010/63/EU of the European Parliament and Council ‘On the protection of animals used for scientific purposes’. The protocol was approved by the ethics committee of the Berlin state authority (‘Landesamt für Gesundheit und Soziales Berlin’, permit number G0294/15). C57BL/6NCrl mice were used for sporozoite infections. All other parasite infections were conducted with SWISS mice.

### 4.3 Generation of recombinant parasite lines

The constructs for endogenous tagging of *FT2* (PBANKA_0931500) were designed for single homologous recombination (Figure S1a). The sequence upstream of the *FT2* stop codon was PCR-amplified from genomic DNA and inserted into the pBAT vector (Kooij, Rauch, & Matuschewski, 2012) using EcoRI and HpaI restriction sites. To introduce the api-GFP marker, the promoter of heat shock protein 70 (*HSP70*, PBANKA_0711900) driving high-level expression of cytoplasmic GFP was replaced with the promoter and amino-terminal sequence of the 20 kDa chaperonin gene (*CPN20*, PBANKA_1347800) using PvuII and PshAI.

The organelle marker vector used for the generation of the WT_mito-mCh/api-GFP_ parasite line was constructed by inserting the promoter and amino-terminal sequence of heat shock protein 70-3 (*HSP70-3*, PBANKA_0914400) into the pBAT-SIL6 vector (Kooij et al., 2012) in frame with mCherry-3xMyc using EcoRI and AgeI, followed by the insertion of the promoter and amino-terminal sequence of *CPN20* in front of GFP using PvuII and PshAI. To generate the *FT2* deletion vectors, 5′ and 3′ flanking regions were PCR-amplified and inserted into the pBAT vector or into the organelle marker vector using SacII in combination with PvuII or NgoMIV, and XhoI in combination with KpnI. *FT2* tagging constructs were linearised with PacI, *FT2* deletion vectors with SalI and NgoMIV and the organelle marker vector with AhdI. *P. berghei* (strain ANKA) WT parasites were transfected with 5 μg of DNA using established protocols (Janse, Franke-Fayard, et al., 2006; Janse, Ramesar, & Waters, 2006; Matz & Kooij, 2015). Isogenic parasite lines were isolated by flow cytometry (Kenthirapalan, Waters, Matuschewski, & Kooij, 2012).

For the generation of the *ft2*^−^_+ft2-tag_ parasite line, pyrimethamine-resistant *ft2*^−^ parasites were first subjected to in vivo negative selection with 5-fluorocytosine, as described previously (Orr, Philip, & Waters, 2012). *ft2*^−^ parasites that had lost their drug-selectable cassette through homologous recombination were isolated by limiting dilution cloning and used as the recipient strain during transfection with the *FT2* complementation construct. This construct was generated by insertion of the *CPN20* promoter sequence into the pBAT-SIL6 vector (Kooij et al., 2012) using SacII and EcoRI, followed by the *FT2* coding sequence which was cloned in frame with mCherry-3xMyc using EcoRI and HpaI. The cytoplasmic GFP expression cassette was removed by restriction digest with PvuII and SfoI and subsequent religation. The plasmid was linearised with AhdI and ApaLI prior to transfection.

Details on the organelle markers, recombination strategies as well as primer combinations and sequences used for cloning and diagnostic PCR can be found in Figures 3a, Figures S1a, S2a, S3a,b, as well as in Table S1.

### 4.4 Subcellular fractionation and immunoblotting

*ft2-tag* blood stage parasites were released from erythrocytes by treatment with 0.15% saponin and were then lysed hypotonically for 1 hr on ice in 10 mM TRIS–HCl, pH 7.5. Lysates were either separated on SDS-polyacrylamide and then transferred onto a nitrocellulose membrane or they were centrifuged 50 min at 100,000*g* and then equal amounts of the soluble and insoluble fractions were transferred directly onto a nitrocellulose membrane. Membranes were probed with rat anti-mCherry (1:1,500; ChromoTek) and mouse anti-HSP70 primary antibodies (1:2,000) (Tsuji, Mattei, Nussenzweig, Eichinger, & Zavala, 1994) in combination with the appropriate horseradish peroxidase-coupled secondary antibodies (1:10,000, JacksonImmunoResearch) facilitating detection by chemiluminescence.

### 4.5 *Plasmodium* life cycle analysis

Asexual blood stage development was analysed with an intravital competition assay (Matz et al., 2013). 500 GFP-fluorescent *ft2*^−^ and 500 mCherry-fluorescent Berred WT blood stage parasites were co-injected intravenously into naïve mice and parasitaemias were analysed daily by flow cytometry. Infected blood containing a total of 1,000 parasites was perpetually transferred into naïve mice after 7 days of infection and parasitaemias were determined by flow cytometry at the day of blood passage.

Experiments involving gametocyte quantification, exflagellation, ookinete cultivation or mosquito infection with individual parasite strains were performed 3 days after intravenous injection of 10^7^ blood stage parasites. Gametocyte conversion was quantified by microscopic analysis of Giemsa-stained thin blood films and expressed as the percentage of mature gametocytes among all blood stages. Exflagellation was quantified by transferring 5 μl of infected blood into 125 μl of RPMI 1640 supplemented with 50 μM xanthurenic acid. Samples were initially incubated at 20°C and then transferred to a counting chamber. Upon detection of the first event, exflagellation centres were quantified microscopically for 6 min with a ×400 magnification. For in vitro fertilisation and ookinete conversion, 10 μl of infected blood were cultivated in 90 μl of RPMI 1640 medium (Thermo Fisher Scientific) supplemented with 10% foetal calf serum (Thermo Fisher Scientific), 50 μM xanthurenic acid (Sigma Aldrich) and 0.85 g/L NaHCO_3_ (pH 8.0) at 20°C. Ookinete cultures were stained live with a mouse anti-P28 antibody (1:500; Sinden et al., 1987) in combination with an Alexa Fluor 568-conugated secondary antibody (1:1,000; Thermo Fisher Scientific) and imaged by fluorescence microscopy. Ookinete conversion was expressed as the percentage of fully developed ookinetes among P28-positive parasites.

*A. stephensi* mosquitoes were allowed to feed on infected mice for 30 min following a 4-hr starvation period. Mosquito oocyst burden was quantified on Day 10 and individual oocysts were analysed at the indicated time points by live fluorescence microscopy of extracted mosquito midguts. For transmission experiments, naïve mice were either injected intravenously with 10,000 sporozoites or subjected to the bites of ≥50 *A. stephensi* mosquitoes which had fed on highly infected mice 21 days earlier. The time of blood stage patency was monitored daily by Giemsa staining or flow cytometry of peripheral blood. A detailed analysis of sporozoite gliding behaviour was confounded by the very low *ft2*^−^ sporulation.

To analyse liver stage development in the absence of *FT2*, an in vivo crossing strategy was used as described previously (Matz, Goosmann, Matuschewski, & Kooij, 2018; Rathnapala, Goodman, & McFadden, 2017). Mixed inocula of 5 x 10^6^ *ft2*^−^ and 5 x 10^6^ Berred WT parasites were injected into naïve mice and fed to *A. stephensi* mosquitoes 3 days later. The sporozoite population resulting from cross-feeding of GFP-fluorescent *ft2*^−^ and mCherry-fluorescent Berred WT parasites was isolated from salivary glands and imaged live to determine the ratios of GFP-, mCherry- and double-fluorescent sporozoites. Three days after transmission to naïve mice, peripheral blood was subjected to flow cytometry to determine the ratios of the individual fluorescent parasite populations of the first blood stage generation. Parasite genomic DNA was extracted from donor and recipient mice for the analysis of parasite genotypes using diagnostic PCR.

The mixed sporozoites were also used to infect hepatocytes in vitro and fluorescent parasites were imaged, quantified and measured 24, 48 and 70 hr following invasion. Analysis of liver-stage development was performed with Huh7 cells, which were seeded onto eight well glass bottom slides (Thermo Fisher Scientific) and infected with 10,000 sporozoites at sub-confluence. The expected fractions for *FT2*-deficient liver stages and emerging blood stages reflect 50% of the observed double-fluorescent sporozoite population (Matz et al., 2018). For a detailed explanation of the underlying genetics, recombination events and calculations, please refer to Figure S4.

### 4.6 Microscopy

All fluorescence imaging was performed with an AxioImager Z2 epifluorescence microscope equipped with an AxioCam MR3 camera (Zeiss). Mutants were passed through the parasite life cycle and imaged live at the indicated time points. Tagged mutant parasites within the parental populations were identified by fluorescence of their cytoplasmic or organellar markers. To distinguish the signal of mCherry-3xMyc-tagged proteins from background fluorescence, Bergreen WT parasites were analysed in parallel. Parasite DNA was visualised with Hoechst 33342 nuclear dye (1:1,000).

For TEM, WT- or *ft2*^−^-infected mosquito midguts were isolated on Day 17 after the blood meal and fixed with 2.5% glutaraldehyde. Midguts were then treated with 1% osmium tetroxide, and further contrasted *en bloc* using 0.5% uranyl acetate. Following dehydration in a graded series of ethanol and propylene oxide, midguts were embedded in epoxy resin and cured at 60°C for at least 24 hr. Sixty nanometre sections were made with a Reichert Ultracut S ultramicrotome (Leica) using a diamond knife. Sections were retrieved on copper slot grids, and stained with 2% uranyl acetate and Reynold’s lead citrate before imaging on an EM 900 TEM (Zeiss) equipped with a wide-angle slow-scan 2K CCD camera (Tröndle Restlichtverstärkersysteme).

### 4.7 Morphological oocyst analysis

Oocysts were imaged live at a focal plane that captured their maximum expansion. Oocysts showing fully or partially developed sporozoites were classified as having entered sporulation. To be classified as containing vesicularisations, oocysts needed to display intracellular accumulations distinct from the remainder of the oocyst cytoplasm, as indicated by DIC imaging. These structures needed to display a rough surface and irregular shape and exclude cytoplasmic fluorescent protein. Fully vesiculated oocysts were also included. These criteria do not apply to frequently observed sporoblast intermediates, which appear as smooth and circular patches beneath the oocyst surface and stain positive for cytoplasmic fluorescent markers.

## Supplementary Material

Supporting information

## Data Availability

The data that support the findings of this study are available from the corresponding author upon request.
