# Supplementary material for: An apicoplast-resident folate transporter is essential for sporogony of malaria parasites
Source: Cell Microbiol. Author manuscript; Available in PMC 2024 Jun 6. (PMC7616068; doi:10.1111/cmi.13266)
Supplement: Supporting information [file EMS196520-supplement-Supporting_information.pdf]

Supplementary information

An apicoplast-resident folate transporter is essential for sporogony of malaria parasites

Francois Korbmacher, Benjamin Drepper, Theo Sanderson, Peer Martin, Thomas Stach, Alexander G. Maier, Kai Matuschewski & Joachim M. Matz

Figure S1 Endogenous tagging of *FT2* in *Plasmodium berghei*

Figure S2 *FT2* deficiency does not impact morphology or integrity of endosymbiotic *Plasmodium* organelles

Figure S3 Ectopic expression of *FT2* restores sporogony in *ft2*⁻ parasites

Figure S4 Inter-chromosomal recombination during the mosquito stage

Table S1 Primer sequences

Supplementary references

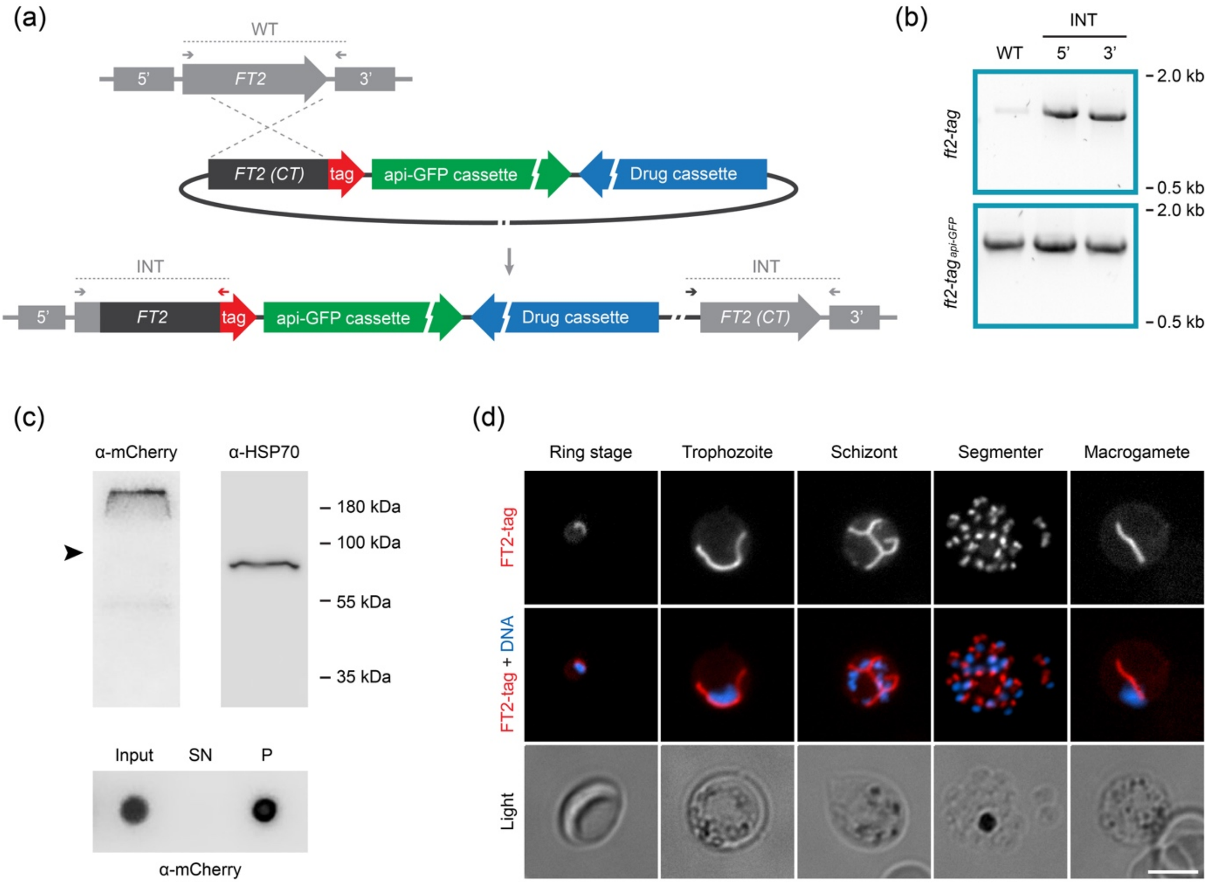

FIGURE S1 Endogenous tagging of *FT2* in *Plasmodium berghei*. (a) Strategy for the generation of transgenic parasites that express the endogenous *FT2* fused to mCherry-3xMyc (tag, red) by single homologous recombination. In addition, recombinant parasites express the drug-selectable hDHFR-yFcu cassette (blue) and a cassette driving expression of cytoplasmic (not shown) or apicoplast-targeted GFP (api-GFP, green, shown), resulting in the *ft2-tag* and *ft2-tag*$_{api\text{-}GFP}$ parasite lines. Wild-type-specific (WT) and integration-specific (INT) primer combinations (Table S1) are indicated by arrows and expected fragments by dotted lines. Shown are the WT locus (top), the transfection vector (middle) and the recombined locus (bottom). (b) Diagnostic PCRs of the mixed parasite populations post-transfection, using the primer combinations indicated in a. Results are shown for the *ft2-tag* (top) and the *ft2-tag*$_{api\text{-}GFP}$ parasite lines (bottom). (c) Subcellular fractionation confirms membrane-association of FT2. Shown are Western blots of *ft2-tag* blood stage parasite lysate using anti-mCherry (top left) and anti-HSP70 primary antibodies (top right). The absence of a band corresponding to the molecular weight of tagged FT2 (arrowhead) is owed to the high degree of membrane insertion causing abnormal separation and transfer behaviour. Dot blot analysis of parasite lysate and of the soluble and membrane fractions confirms expression of membrane-associated FT2-tag (bottom). (d) Live fluorescence microscopy reveals that FT2 localizes to a dividing organelle during blood stage development. Shown are the fluorescent signal of tagged

FT2 (red, 1st row), a merge with Hoechst 33342 nuclear stain (DNA, blue, 2nd row), and transmitted light images (3rd row). Bar, 5 μm

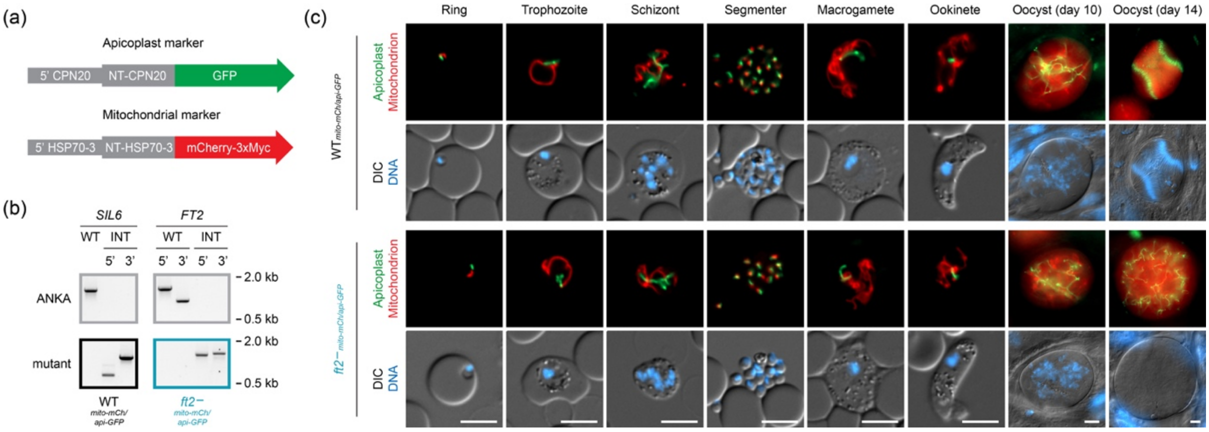

FIGURE S2 *FT2* deficiency does not impact morphology or integrity of endosymbiotic *Plasmodium* organelles. (a) Schematic representation of the marker cassettes used for the generation of the WT$_{mito-mCh/api-GFP}$ and *ft2*$^{-}_{mito-mCh/api-GFP}$ parasite lines. The apicoplast marker cassette (api-GFP) consists of the promoter (5') and amino-terminal (NT) sequence of the 20 kDa chaperonin CPN20 fused to GFP. The mitochondrial marker cassette (mito-mCherry) consists of the 5' and NT sequence of heat shock protein 70-3 (*HSP70-3*) fused to mCherry-3xMyc (Matz et al., 2018). Marker cassettes were integrated together into the silent intergenic locus on *P. berghei* chromosome 6 (*SIL6*) to generate the WT$_{mito-mCh/api-GFP}$ reference line, or into the endogenous *FT2* locus upon gene deletion to yield the *ft2*$^{-}_{mito-mCh/api-GFP}$ parasite line. (b) Diagnostic PCRs of *SIL6* and of the *FT2* locus in WT (ANKA, top) and in the isolated WT$_{mito-mCh/api-GFP}$ and *ft2*$^{-}_{mito-mCh/api-GFP}$ parasite lines (bottom). Wild-type (WT) and integration-specific primer combinations (INT) have been used as indicated in Table S1. (c) Shown are representative live fluorescence micrographs of WT$_{mito-mCh/api-GFP}$ and *ft2*$^{-}_{mito-mCh/api-GFP}$ parasites during life cycle progression, including the merged signals of api-GFP (green) and mito-mCherry (red, top) as well as a merge of differential interference contrast images (DIC) with Hoechst 33342 nuclear stain (DNA, blue, bottom). Bars, 5 µm.

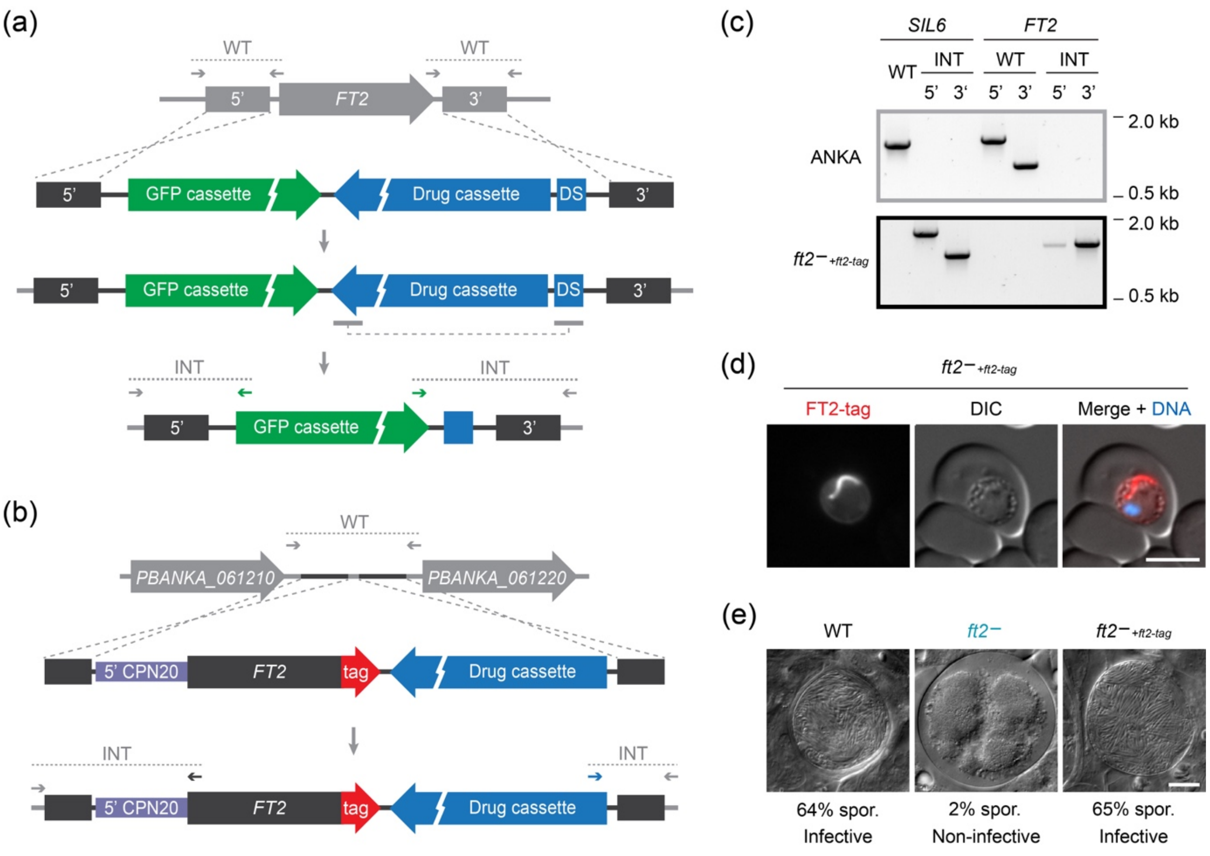

FIGURE S3 Ectopic expression of *FT2* restores sporogony in *ft2*$^{-}$ parasites. (a, b) Recombination strategy for complementation of *ft2*$^{-}$ parasites with tagged *FT2*. (a) Endogenous *FT2* was targeted with a replacement plasmid containing the 5' and 3' regions flanking the open reading frame. Upon positive selection, pyrimethamine-resistant *ft2*$^{-}$ parasites harbour the drug-selectable hDHFR-yFcu cassette (blue) and a high-expressing GFP-cassette (green). Negative selection of *ft2*$^{-}$ parasites with 5-fluorocytosine removes large parts of the drug-selectable cassette through homologous recombination of a duplicated sequence (DS), yielding pyrimethamine-sensitive *ft2*$^{-}$ parasites. (b) The silent intergenic locus on *P. berghei* chromosome 6 (*SIL6*) of the drug cassette-recycled *ft2*$^{-}$ line was targeted with a plasmid containing the promoter of the 20 kDa chaperonin (5' CPN20, purple) driving expression of mCherry-3xMyc-tagged (tag, red) *P. berghei FT2.* The construct also contained the drug-selectable cassette for positive selection with pyrimethamine. Shown are the WT loci (top), the transfection vectors (middle) and the recombined loci before and after drug cassette removal (bottom). Primer combinations specific for the WT loci and for integration (INT) are indicated by arrows and expected amplicons by dotted lines. (c) Shown are diagnostic PCRs of *SIL6* and of the endogenous *FT2* locus in WT (ANKA) and in the isolated *ft2*$^{-}_{+ft2\text{-}tag}$ parasite line. Primer combinations have been used as indicated in a and b and in Table S1. (d) Localization of ectopically expressed FT2. Shown is a live fluorescence micrograph of a

representative *ft2*$^{-}$$_{+ft2\text{-}tag}$ trophozoite, including the fluorescent signal of tagged FT2 (red, left), differential interference contrast (DIC, middle) and a merge including Hoechst 33342 nuclear stain (DNA, blue, right). Bar, 5 µm. (e) Phenotypic rescue of *ft2*$^{-}$ parasites by ectopic expression of tagged *FT2*. Shown are representative DIC images of WT (left), *ft2*$^{-}$ (middle) and *ft2*$^{-}$$_{+ft2\text{-}tag}$ oocysts 17 days after the blood meal. The occurrence of sporulating oocysts (spor.) and ability to establish patent blood infection in mice upon inoculation with salivary gland-associated sporozoites are indicated below. Data are derived from one feeding experiment. All mice injected with *ft2*$^{-}$ sporozoites remained blood stage negative, all WT and *ft2*$^{-}$$_{+ft2\text{-}tag}$-injected mice developed patent blood infection. n = 3 mice. Bar, 10 µm.

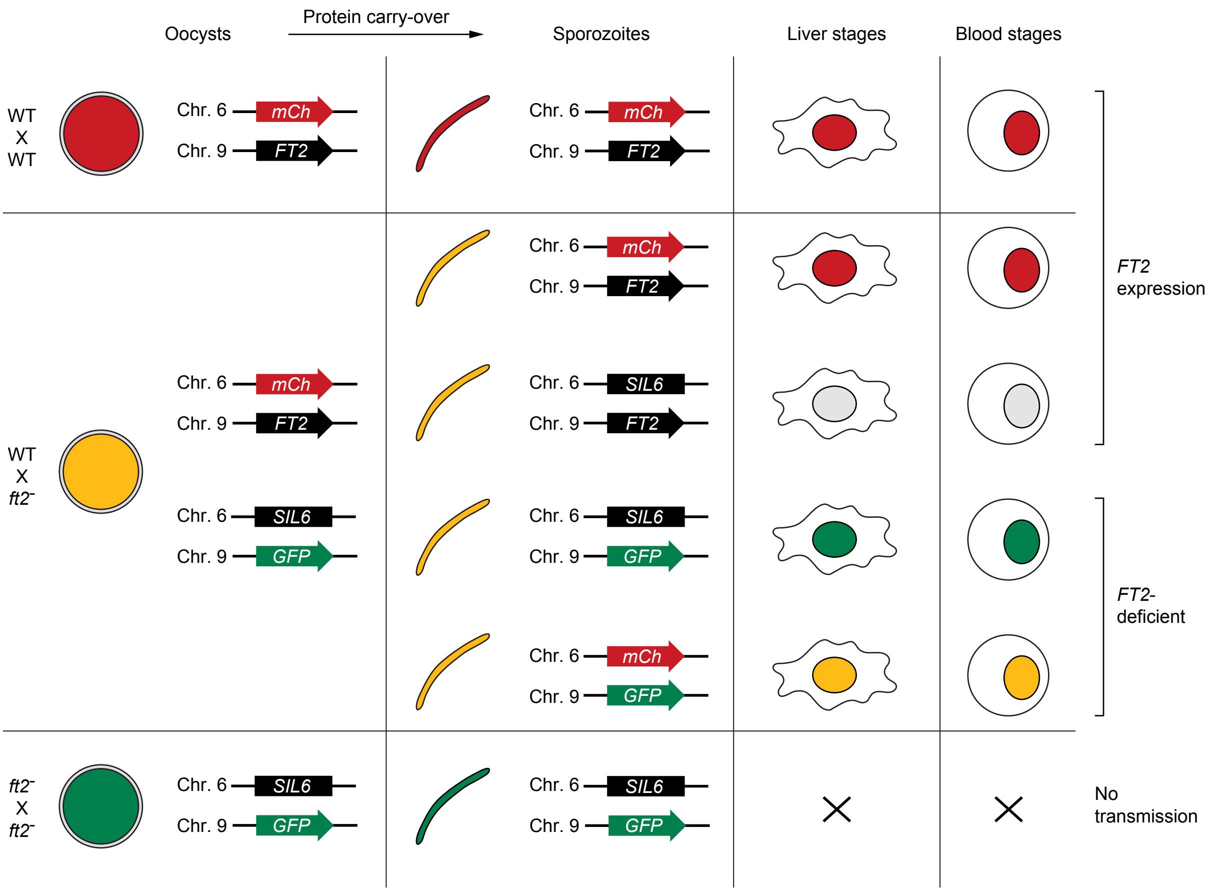

FIGURE S4 Inter-chromosomal recombination during the mosquito stage. Genotypes and fluorescence properties of homozygous Berred WT (red, top), heterozygous Berred WT x *ft2*⁻ oocysts (yellow, middle) and homozygous *ft2*⁻ oocysts (green, bottom), and of the resultant sporozoites, liver stages and blood stages. Depicted are the *FT2* locus on chromosome (Chr.) 9 and the silent intergenic locus on chromosome 6 (*SIL6*). Berred WT parasites harbour the intact *FT2* gene and express mCherry (mCh) from *SIL6*. *ft2*⁻ parasites express GFP from the disrupted *FT2* locus, while *SIL6* is unaltered. Inter-chromosomal recombination during mosquito-stage development yields four distinct genotypes for the haploid sporozoites emerging from heterozygous oocysts. Note that these sporozoites remain double-fluorescent due to protein carry-over from the oocyst. Sporozoites from homozygous *ft2*⁻ oocysts are non-infective. For the quantitative analysis of mosquito-to-mouse transition, only fluorescent parasites were quantified. Thus, non-fluorescent liver and blood stages were not detected, but accounted for in the calculation of the expected values.

Table S1. Primer sequences.

Primer Name	Primer Sequence (restriction sites)	WT (bp)[a]	INT (bp)[b]	Use[c]	Target	Reference
5'-PbFT2-F-SacII	aataatccgcggccaacatttccatctcgcg	1,069		TV	5' Pb*FT2*	This study
5'-PbFT2-R-PvuII	tttaatcagctggtatgtaactattcgtatgtgcg			TV	5' Pb*FT2*	This study
5'-PbFT2-F-NgoMIV	aattatgccggcccaacatttccatctcgcg	1,069		TV	5' Pb*FT2*	This study
5'-PbFT2-R-SacII	attaatccgcgggtatgtaactattcgtatgtgcg			TV	5' Pb*FT2*	This study
3'-PbFT2-F-XhoI	aataatctcgagaaaaaatacagaaatgagcggc	682		TV	3' Pb*FT2*	This study
3'-PbFT2-R-KpnI	aataatggtacctaaacggtttggatgaagcg			TV	3' Pb*FT2*	This study
CT-PbFT2-F-EcoRI	aattgtgaattcttggaatgcgaagaaaaccg	1,079		TV	CT Pb*FT2*	This study
CT-PbFT2-R-HpaI	tttttgttaactttctgtattttttatttttaaactttcaatttg		1,386	TV	CT Pb*FT2*	This study
NT-PbFT2-F-EcoRI	taatgagaattcaaaatgattgagaaatcgaataacatatatc			TV	NT Pb*FT2*	This study
5'-CPN20-F-PvuII	gctaagccagctgaaaaaactaacaatatttatttgacgc	1,361		TV	5' Pb*CPN20*	This study
NT-CPN20-R-PshAI	actcacagacatatgtcctctaattgttctattatctagc			TV	NT Pb*CPN20*	This study
5'-CPN20-F-SacII	attaatccgcgggtcctattctctcaacgatgg	1,085		TV	5' Pb*CPN20*	This study
5'-CPN20-R-EcoRI	aaaagtgaattcgcataaatatttattgtatattcatatagtttag			TV	5' Pb*CPN20*	This study
5'-HSP70-3-F-EcoRI	aaaaaagaattctatggaggggactgttgatgg	2,008		TV	5' Pb*HSP70-3*	This study
NT-HSP70-3-R-AgeI	aaattaaccggtagcatcttcatcaaattttctacc			TV	NT Pb*HSP70-3*	This study
5'-PbFT2-F	gtttcttttacctgtttcttttaccc	1,376	1,428/1,807	GT	5' Pb*FT2*	This study
5'-PbFT2-R	aatttatcaaaggcaattcttctcc			GT	5' Pb*FT2*	This study
3'-PbFT2-F	ggctccctcacaaaaaaggg	926		GT	3' Pb*FT2*	This study
3'-PbFT2-R	tgcccgaaaaatagaagtccc		1,377	GT	3' Pb*FT2*	This study
CT-PbFT2-F	gattaatacatttagcatctttggc	1,342	1,318	GT	CT Pb*FT2*	This study
CT-PbFT2-R	attggagcatttttttgcgc		2,011	GT	CT Pb*FT2*	This study
SIL6F	gacagcgcatatgatggatg	1,315	1,804	GT	Pb*SIL6*	Kenthirapalan et al., 2012
SIL6R	tacgaatacgcaatttctcaaac		1,247	GT	Pb*SIL6*	Kenthirapalan et al., 2012
5'DHFRrev	atgaaataccgctccattttcc			GT	5' Pb*DHFR-TS*	Kenthirapalan et al., 2012
5'HSP70rev	caatttgttgtacataaaataggcag			GT	5' Pb*HSP70*	Kenthirapalan et al., 2012
3'DHFS-F	gcttttcgctatatcgctatc			GT	3' Pb*DHFS-FPGS*	Kooij et al., 2012
5'-PbHSP70-3-R	catgataacccattactaatatgtgg			GT	5' Pb*HSP70-3*	This study
mCherryRev	ccctccatgtgaaccttgaag			GT	*mCherry*	Haussig et al., 2011
M13R	caggaaacagctatgaccatg			GT	*M13*	Haussig et al., 2011

[a] Sizes of the PCR products of forward and reverse primers on WT genomic DNA.

[b] Sizes of the respective integration-specific PCR products; forward 5' gene-specific primers and carboxy-terminal tagging-specific forward primers combined with 5'HSP70rev (*ft2*⁻), 5'-PbHSP70-3-R ($ft2^{-}_{api\text{-}GFP/mito\text{-}mCh}$), 5'-PbFT2-R ($ft2^{-}_{+ft2\text{-}tag}$) or mCherryRev (tagged lines), and reverse 3' gene-specific primers combined with 5'DHFRrev ($ft2^{-}$ and $ft2^{-}_{api\text{-}GFP/mito\text{-}mCh}$), 3'DFHS-F ($ft2^{-}_{+ft2\text{-}tag}$) or M13R (tagged lines).

[c] Primers used for construction of Transfection Vectors (TV), for GenoTyping (GT), or quantitative real-time PCR (qPCR). Used primer combinations are indicated in Figures 3a, S1a and S3 a and b.

SUPPLEMENTARY REFERENCES

Haussig, J. M., Matuschewski, K., & Kooij, T. W. (2011). Inactivation of a *Plasmodium* apicoplast protein attenuates formation of liver merozoites. *Mol Microbiol, 81*(6), 1511-1525.

Kenthirapalan, S., Waters, A. P., Matuschewski, K., & Kooij, T. W. (2012). Flow cytometry-assisted rapid isolation of recombinant *Plasmodium berghei* parasites exemplified by functional analysis of aquaglyceroporin. *Int J Parasitol, 42*(13-14), 1185-1192.

Kooij, T. W., Rauch, M. M., & Matuschewski, K. (2012). Expansion of experimental genetics approaches for *Plasmodium berghei* with versatile transfection vectors. *Mol Biochem Parasitol, 185*(1), 19-26.
